# Structural cerebellar reserve positively influences outcome after severe stroke

**DOI:** 10.1093/braincomms/fcac203

**Published:** 2022-08-04

**Authors:** Fatemeh Sadeghihassanabadi, Benedikt M Frey, Winifried Backhaus, Chi-un Choe, Simone Zittel, Gerhard Schön, Marlene Bönstrup, Bastian Cheng, Götz Thomalla, Christian Gerloff, Robert Schulz

**Affiliations:** Department of Neurology, University Medical Center Hamburg-Eppendorf, 20246 Hamburg, Germany; Department of Neurology, University Medical Center Hamburg-Eppendorf, 20246 Hamburg, Germany; Department of Neurology, University Medical Center Hamburg-Eppendorf, 20246 Hamburg, Germany; Department of Neurology, University Medical Center Hamburg-Eppendorf, 20246 Hamburg, Germany; Department of Neurology, University Medical Center Hamburg-Eppendorf, 20246 Hamburg, Germany; Institute of Medical Biometry and Epidemiology, University Medical Center Hamburg-Eppendorf, 20246 Hamburg, Germany; Department of Neurology, University Medical Center Hamburg-Eppendorf, 20246 Hamburg, Germany; Department of Neurology, University Medical Center Leipzig, 04103 Leipzig, Germany; Department of Neurology, University Medical Center Hamburg-Eppendorf, 20246 Hamburg, Germany; Department of Neurology, University Medical Center Hamburg-Eppendorf, 20246 Hamburg, Germany; Department of Neurology, University Medical Center Hamburg-Eppendorf, 20246 Hamburg, Germany; Department of Neurology, University Medical Center Hamburg-Eppendorf, 20246 Hamburg, Germany

**Keywords:** reserve, capacity, volumetry, cerebellum, lobules

## Abstract

The concept of brain reserve capacity positively influencing the process of recovery after stroke has been continuously developed in recent years. Global measures of brain health have been linked with a favourable outcome. Numerous studies have evidenced that the cerebellum is involved in recovery after stroke. However, it remains an open question whether characteristics of cerebellar anatomy, quantified directly after stroke, might have an impact on subsequent outcome after stroke. Thirty-nine first-ever ischaemic non-cerebellar stroke patients underwent MRI brain imaging early after stroke and longitudinal clinical follow-up. Structural images were used for volumetric analyses of distinct cerebellar regions. Ordinal logistic regression analyses were conducted to associate cerebellar volumes with functional outcome 3–6 months after stroke, operationalized by the modified Rankin Scale. Larger volumes of cerebellar lobules IV, VI, and VIIIB were positively correlated with favourable outcome, independent of the severity of initial impairment, age, and lesion volume (*P* < 0.01). The total cerebellar volume did not exhibit a significant structure-outcome association. The present study reveals that pre-stroke anatomy of distinct cerebellar lobules involved in motor and cognitive functioning might be linked to outcome after acute non-cerebellar stroke, thereby promoting the emerging concepts of structural brain reserve for recovery processes after stroke.

## Teaser

Sadeghihassanabadi et al. report a significant association between larger volumes of specific cerebellar lobules of the motor and cognitive domains, obtained acutely after non-cerebellar stroke, and subsequent favourable outcome after 3–6 months. These data promote the emerging concepts of structural brain reserve for recovery processes after stroke.

## Graphical Abstract

**Graphical abstract:**
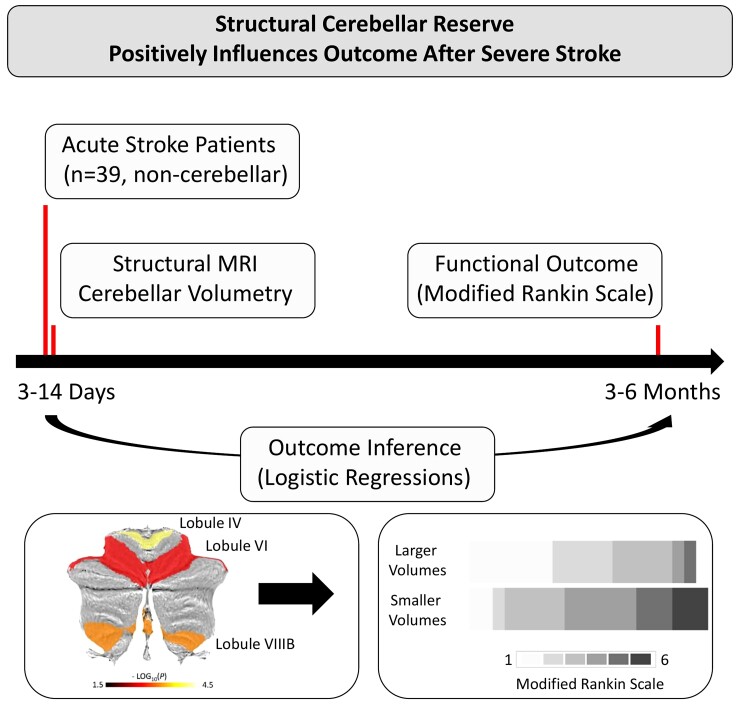

## Introduction

The concept of brain reserve capacity has been increasingly recognized in stroke recovery research. According to this concept, reserve is a relevant feature of brain structure or function that moderates the relationship between brain pathology or injury and their clinical manifestation.^1,2^ Recent studies have provided novel insights on how baseline, i.e. pre-stroke, surrogates of brain health, obtained directly after stroke, can be used to inform correlative or predictive outcome models to understand inter-subject variability in stroke recovery better. For instance, global structural measures such as overall brain atrophy^3,4^ or white matter hyperintensities burden^5^ could be related to unfavourable outcome after stroke. Spatial specificity and mechanistic insights regarding specific brain regions have been recently developed by a study involving severely impaired stroke patients. The authors reported that larger thickness of specific contralesional cortices at baseline was associated with better outcome.^6^ Importantly, cortical brain areas have been consistently set in the focus of structural^7^ and functional^8^ neuroimaging studies. More recently, the cerebellum and its cortico-cerebellar interactions have gained increasing interest as well. Densely connected to multiple motor- and non-motor areas,^9^ the cerebellum forms an important hub in the human sensorimotor network. Supratentorial strokes can critically impact cerebellar neuronal activity and lead to cerebellar hypometabolism, hypoperfusion, and atrophy of the contralesional cerebellum,^10-13^ which have been considered as key features of cerebellar diaschisis.^14^ Evidence from brain activation,^8,15,16^ functional connectivity,^17-19^ and structural imaging studies^20,21^ convergingly indicates that the cerebellum significantly contributes to recovery processes, residual motor functions, and treatment gains in stroke patients. In continuation, non-invasive brain stimulation studies have suggested that the cerebellum might be an innovative target to influence the cerebello-cortical plasticity and subsequently to promote recovery.^22-25^ So far, the concept of cerebellar reserve has been primarily developed for cerebellar pathology such as cerebellar stroke or neurodegenerative diseases.^26,27^ To what extent this concept holds true for recovery aspects and functional outcome of non-cerebellar ischaemic strokes however has not been explored systematically.

The present study aimed at investigating whether cerebellar anatomy, quantified directly after first-ever unilateral ischaemic stroke, might show associations with subsequent outcome. We hypothesized that particularly volumes of motor-related regions such as lobules I–VI and VIII^28^ would show structure-outcome relationships. We re-analysed structural imaging and clinical data taken from two independent cohorts.^29,30^ The present work is in continuation of our previous study which focused on the structural reserve of contralesional cortices to promote favourable outcome after stroke.^6^

## Materials and methods

### Cohort and clinical data

The data set incorporates two independent cohorts of acute stroke patients from previously published observational studies. Cohort 1 (C_1_) comprised 61 acute ischaemic stroke patients admitted to the University Medical Center Hamburg-Eppendorf who were recruited between 2012 and 2017.^30^ Cohort 2 (C_2_) consisted of 30 more severely impaired acute stroke patients, admitted to the same medical centre from 2017 to 2020.^29^ In brief, inclusion criteria for both studies were as follows: first-ever unilateral ischaemic stroke, upper extremity motor deficit involving hand function, no history of previous neurological or psychiatric illness, age ≥18 years. Acute stroke patients underwent structural MRI in the first days after the event as time point T_1_ (C_1_: days 3–5, C_2_: days 3–14). Follow-up time point T_2_ was defined in the late subacute stage of recovery after three months,^31^ or, in cohort C_2_ in which clinical data for this time point were not available, after 6 months.^29^ For proper integration of these two patient groups, only patients of C_1_ were further considered in the present analysis who met the initial inclusion criteria of C_2_, i.e. modified Rankin Scale (MRS) T_1_ > 3 or Barthel index (BI) of ≤ 30. As the result, the final sample consisted of 39 more severely impaired patients. Neurological symptom burden at T_1_ was operationalized via the National Institutes of Health Stroke Scale (NIHSS). MRS at follow-up T_2_ was considered as the functional outcome. All participants provided informed consent themselves or via a legal guardian, following the ethical Declaration of Helsinki. The studies were approved by the local ethics committee.

### Brain imaging

For both data sets, a 3T Skyra MRI scanner (Siemens, Erlangen, Germany) equipped with a 32-channel head coil was used to obtain structural high-resolution T1-weighted images applying a 3-dimensional magnetization-prepared rapid gradient echo sequence [repetition time (TR) = 2500 ms, echo time (TE) = 2.12 ms, flip angle 9°, 256 coronal slices with a voxel size of 0.8 × 0.8 × 0.9 mm³, field of view (FOV) = 240 mm]. T2-weighted images were also acquired by using a fluid-attenuated inversion recovery sequence (TR = 9000 ms, TE = 86 ms, TI = 2500 ms, flip angle 150°, 43 transversal slices with a voxel size of 0.7 × 0.7 × 3.0 mm³, FOV = 230 mm) for stroke lesion delineation. Data sets were processed with volBrain^32^ and the CERES pipeline for cerebellum lobule segmentation and volumetric analysis.^33^ Processing steps include de-noising, inhomogeneity correction, linear registration to Montreal Institute of Neurology (MNI) space, cropping of the cerebellum, non-linear registration to an MNI cerebellum template, intensity normalization, and a non-linear registration to a subject-specific library. Cerebellar structures were then outlined according to published definitions.^34^ Volumes were estimated for 13 cerebellar regions, including lobules I–II, III, IV, V, VI, VIIB, VIIIA, VIIIB, IX, X, crus I, and crus II. The total volume of the cerebellum and the total intracranial volume (ICV) were also determined.

### Statistics

Statistical analyses were conducted using R version 4.0.3.^35^ Ordinal logistic regression analyses (function *polr* from the MASS package),^36^ were carried out to relate volume estimates obtained at T_1_ to MRS at T_2_. In line with previous structural imaging studies,^6,37^ the patients’ group was split by the median into two subsets per volume (larger and smaller regional volumes compared to the median) to improve statistical power. Regression models were fit across the entire group of patients, one for each of the 13 cerebellar volumes of interest which were treated as independent variables of interest. Lesion volume at time point T_1_, age, and ICV were treated as additional independent variables. Given relevant collinearities between volume estimates and age and ICV, respectively, these two covariates were included after linear residualization against the regional volumes.^6^ Model results are presented without and with adjustment for neurological symptom burden at T_1_ (NIHSS). Odds ratios (ORs) to score higher on MRS at T_2_ are given for the cerebellar volumes (reference: larger volume group) with corresponding 95% confidence intervals and *P* values. Herein, OR values below 1 would indicate a lower risk of a worse outcome for patients with larger volume estimates when compared to patients with smaller volumes. Lesion volumes were LOG_10_-transformed to improve data distribution. Leave-one-out model analysis (LOOA) was used to probe the robustness of the significant findings. Statistical significance was assumed at a *P* value < 0.05.

### Data availability

Data will be made available by the authors upon reasonable request.

## Results

### Demographic and clinical data

Table 1 shows the individual demographic and clinical data. The data used for analysis included 39 acute stroke patients [18 females, 23 right-sided strokes, 2 left-handed, 5 infratentorial strokes, median age 74 years, interquartile range (IQR) 64–79]. The median NIHSS score at T_1_ was 9 (6–13), the median MRS at T_1_ was 4 (4–5), at T_2_ 3 (2–4). Median lesion volume was 26.7 mL (6.2–77.5 mL).

**Table 1:** Demographic and clinical data

ID	Cohort	Age	Sex	Side	Location	Volume, mL	MRS T_1_	NIHSS T_1_	MRS T_2_	TP
1	C1	43	M	R	S	79.8	4	13	2	3
2	C1	56	M	R	S	2.5	4	13	2	3
3	C1	49	F	L	S	53.8	5	10	2	3
4^a^	C1	69	M	R	S	25.1	4	3	1	3
5	C1	73	F	R	S	26.8	4	3	1	3
6	C1	58	M	R	I	0.7	4	7	2	3
7	C1	73	F	L	S	5.8	4	9	4	3
8	C1	50	M	R	S	25.5	4	7	2	3
9	C1	77	F	R	S	9.1	5	8	4	3
10^a^	C1	65	M	L	S	6.6	4	8	3	3
11	C1	85	F	R	S	16.7	4	7	4	3
12	C1	81	M	L	I	0.6	4	4	1	3
13	C1	81	M	L	S	1.7	4	4	2	3
14	C1	76	M	L	I	1.7	4	5	1	3
15	C1	48	M	L	S	24.4	4	7	1	3
16	C1	87	F	L	S	1.0	4	1	1	3
17	C1	47	M	R	S	2.6	4	6	3	3
18	C1	50	M	R	S	50.1	4	4	1	3
19	C1	83	F	L	I	3.3	4	5	3	3
20	C2	78	M	L	S	58.1	5	17	5	6
21	C2	83	F	L	S	101.4	5	20	6	3
22	C2	76	M	R	S	101.0	5	11	3	3
23	C2	63	M	L	S	55.8	4	13	1	3
24	C2	71	M	R	S	75.2	5	15	6	3
25	C2	73	F	L	S	14.4	4	9	3	3
26	C2	77	F	R	S	286.7	4	11	4	6
27	C2	71	F	R	S	38.4	5	9	3	6
28	C2	80	F	L	S	20.5	5	11	4	6
29	C2	58	M	R	S	98.0	5	13	5	3
30	C2	67	F	R	S	7.4	4	11	3	6
31	C2	80	M	R	S	108.4	5	16	6	3
32	C2	79	F	R	S	120.4	5	8	3	6
33	C2	85	F	R	S	33.5	5	15	5	3
34	C2	78	M	R	S	178.1	5	17	4	3
35	C2	73	F	R	S	27.6	4	5	1	3
36	C2	76	M	R	S	91.8	5	15	4	3
37	C2	78	F	L	S	33.6	4	10	3	3
38	C2	74	M	L	S	303.3	5	24	5	6
39	C2	89	F	R	I	2.6	5	7	3	3

^a^ All patients were right-handed except IDs 4 and 10. TP (time point) at T_2_ indicates whether clinical follow-up data were available after three or six months after stroke. Side indicates the affected hemisphere; R, right or L, left; S, supratentorial lesion location; I, infratentorial lesion location.

### Regional cerebellar brain volume and outcome after stroke

**Figure 1:**
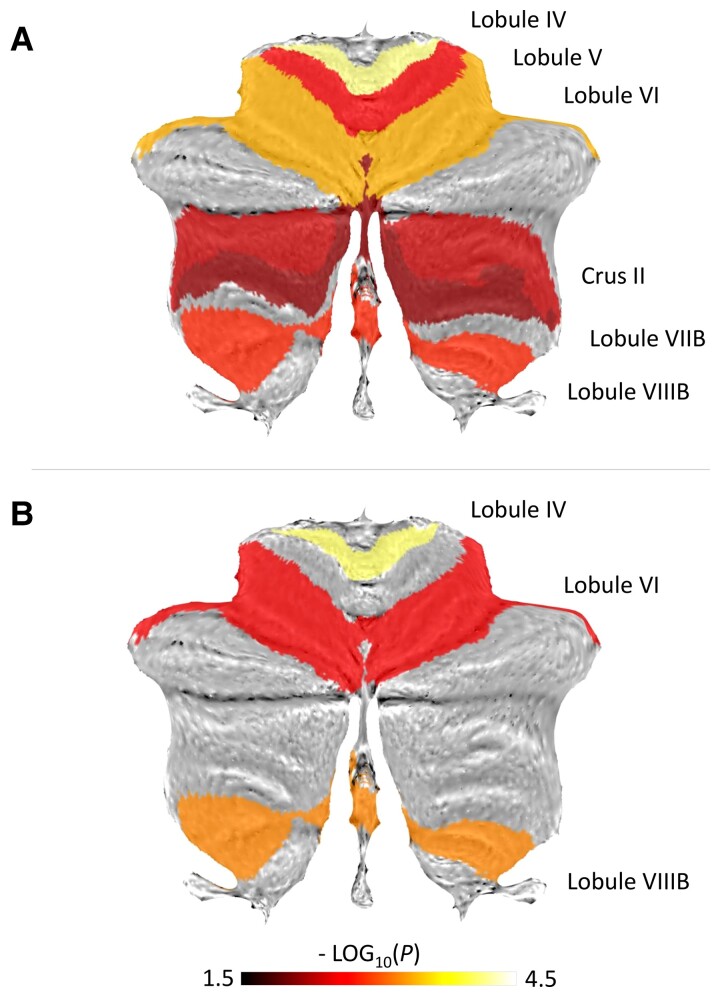
**Cerebellar brain volumes and outcome after stroke.** Labels exhibiting significant association between regional volume and MRS at T_2_ are visualized on a cerebellar flatmap template with colours indicating-log10(*P*). **A** Regression results without adjustment for the initial clinical deficit. **B** Regression results with adjustment. *P* values (uncorrected) are derived from individual regression models

Structure-outcome relationships of cerebellar brain volumes were explored using ordinal logistic regression models. Without adjustment for the initial deficit, we found positive associations not only for the total cerebellar volume but also for distinct lobules related to motor functions (IV, V and VI, VIIIB) and cognitive functions (VIIB, crus II). Specifically, patients with larger volumes in these areas compared to the median showed significantly reduced probability of scoring one level worse in MRS at follow-up compared to patients with smaller volumes (Fig. 1A, Table 2). Numerically, the probability of better outcome was particularly influenced by volumes of the motor-related lobules IV and VI.

**Table 2:** Cerebellar brain volumes and outcome after stroke (not adjusted for the initial deficit)

Region	OR (95% CI)	*P*
**Cerebellum**	**0.16** (**0.04–0.56)**	**0**.**004**
Lobule I-II	0.79 (0.25**–**2.53)	0.695
Lobule III	0.5 (0.15**–**1.58)	0.240
**Lobule IV**	**0.09** (**0.02–0.33)**	**<0**.**001**
**Lobule V**	**0.20** (**0.05–0.66)**	**0**.**008**
**Lobule VI**	**0.12** (**0.03–0.45)**	**0**.**001**
Crus I	0.31 (0.08**–**1.04)	0.059
**Crus II**	**0.21** (**0.05–0.72)**	**0**.**013**
**Lobule VIIB**	**0.25** (**0.07–0.83)**	**0**.**023**
Lobule VIIIA	0.31 (0.09**–**1.02)	0.054
**Lobule VIIIB**	**0.17** (**0.04–0.60)**	**0**.**005**
Lobule IX	0.48 (0.15**–**1.5)	0.207
Lobule X	0.48 (0.15**–**1.51)	0.209

Regions exhibiting a significant association between volume and outcome are highlighted in bold. Results are adjusted for age, lesion volume, and ICV. ORs with 95% CIs are given for patients with larger volumes (reference) of rising one level in MRS compared to patients with smaller volumes. *P* values are uncorrected. Results are ordered by region.

Focussing on stroke recovery, NIHSS at T_1_ was additionally included in these models. After adjustment, structure-outcome associations remained significant for lobules IV, VI, and VIIIB (Fig. 1B, Table 3). Of note, volumes for these three lobules were not directly associated with NIHSS at T_1_ (all *P*≥0.06). Conversely, lesion volume was positively related to NIHSS at T_1_, corrected for age and ICV (*P* < 0.001). Sensitivity analyses were conducted to further explore the robustness of these findings. Results remained significant when excluding five patients with infratentorial strokes (all *P*≤0.01) or when excluding all patients with MRS = 6 (dead) at T_2_ (*P*≤0.02).

**Table 3:** Cerebellar brain volumes and outcome after stroke (adjusted for the initial deficit)

Region	OR (95% CI)	*P*
Cerebellum	0.39 (0.10**–**1.47)	0.165
Lobule I-II	1.02 (0.28**–**3.85)	1.000
Lobule III	0.5 (0.14**–**1.71)	0.266
**Lobule IV**	**0.08** (**0.02–0.33)**	**<0**.**001**
Lobule V	0.23 (0.06**–**0.82)	0.024^a^
**Lobule VI**	**0.15** (**0.03–0.62)**	**0**.**008**
Crus I	0.74 (0.18**–**3.03)	0.678
Crus II	0.43 (0.11**–**1.6)	0.208
Lobule VIIB	0.48 (0.13**–**1.68)	0.252
Lobule VIIIA	0.57 (0.15**–**2.09)	0.392
**Lobule VIIIB**	**0.12** (**0.03–0.47)**	**0**.**002**
Lobule IX	0.29 (0.08**–**1.02)	0.054
Lobule X	0.32 (0.08**–**1.1)	0.069

Regions exhibiting a significant association between volume and outcome are highlighted in bold. Results are adjusted for age, lesion volume, ICV, and the initial deficit. ORs with 95% CIs are given for patients with larger volumes (reference) of rising one level in MRS compared to patients with smaller volumes. *P* values are uncorrected.

^a^ Indicates that this finding is not significant after LOOA (*P* = 0.07). Results are ordered by region.

## Discussion

The main finding of the present study was that characteristics of cerebellar anatomy, obtained directly after stroke, are significantly associated with outcome after severe ischaemic stroke. Specifically, we found that larger volumes of cerebellar lobules IV, VI, and VIIIB were positively correlated with favourable outcome, independent of the degree of initial impairment, age, and lesion volume. The total cerebellar volume did not exhibit a significant structure-outcome relationship. These results extend previous data regarding cerebellar reserve capacity in cerebellar pathology by showing that the structural state of distinct, functionally defined cerebellar lobules in the motor and cognitive domain might also contribute to outcome after acute non-cerebellar stroke and thereby promoting the emerging concepts of structural brain reserve for recovery processes after stroke.

This study is founded on the broad body of literature indicating that the cerebellum is significantly involved in recovery after stroke. For instance, brain activation studies have found that a lateralized, back-to-normal cerebellar activation correlated with good motor performance.^8,15,16^ Functional connectivity studies have reported increases in cortico-cerebellar coupling during spontaneous recovery or neurorehabilitative training^17-19^ One study in pontine stroke patients has reported the occurrence of functional remapping particularly in the subcortical-cerebellum network.^38^ Structural imaging studies have associated preserved integrity of cortico-cerebellar motor pathways to cortical excitability^20^ and residual motor functions^21^ in chronic stroke patients. For structure-outcome inference over time, available data are remarkably limited. One study found that the amount of grey matter volume decreases of the anterior cerebellar lobe within the first 6 months after stroke inversely correlated with the extent of functional improvement.^39^ Extending these longitudinal findings, the present study reveals that baseline, i.e. pre-stroke anatomy, quantified early after stroke might contain relevant information regarding inter-subject variability in the subsequent outcome. Herein, the region-wise analysis revealed that risk reduction for scoring higher on the MRS at T_2_ in patients with larger cerebellar volumes was not an overall feature of the cerebellum, rather it primarily attributed to distinct motor-related cerebellar lobules IV, VI, and VIIIB. Previous studies have revealed somatotopic representations of the body in these lobules with an upside-down map in the anterior cerebellum along with a second representation in lobule VIII.^28,40^ Tracing studies have shown cortico-cerebellar circuits originating in the motor cortex and targeting lobules IV, V, and VI.^41^ Diffusion-tensor imaging and tractography in humans have found evidence of reciprocal cortico-cerebellar tracts between lobules V and VI and the primary motor cortex and the dorsal premotor cortex.^42^ Notably, brain activation patterns in the latter two areas, both on the ipsi- and contralesional hemispheres, have been convergingly linked with recovery processes after stroke.^8,43,44^ In healthy aging, regional grey matter volumes in lobules IV–VI have been associated with motor functions.^45^

Therefore, and as a first speculative interpretation, the present structure-outcome associations detected for lobules IV and VI might indicate that the cerebellum with larger structural/functional reserve in these regions drives neuroplastic adaptive processes via cerebello-cortical connectivity^27^ to compensate for impaired motor output.

In addition to the functional importance in the motor domain, lobule VI has also been reported to be involved in attentional and executive processing and visuospatial working memory tasks with functional connectivity to fronto-parietal networks.^28^ This contribution of anatomy of lobule VI of the cognitive domain is well in line with the upregulation phenomenon seen in cognition-related cortical networks after stroke.^46,47^ Such an upregulation has been explained by attentional processes to motor performance^48^ or by motor learning strategies,^49^ particularly early after stroke and in patients with more severe motor deficits. In fact, motor learning in healthy participants has been reported to engage various regions of the cerebellum, including anterior regions i.e. lobules IV, V and VI and VIIIA/B.^28,50-52^ Therefore, an alternative interpretation might be that larger volumes of these lobules might parallel larger reserve capacity for motor learning strategies, contributing to re-learning of lost motor functions. Of note, meta-analyses have shown that lobule V and VI activate during motor learning paradigms. However, only lobule VI activation remains stable when rather simple motor execution demands are regressed out.^53^ This might also explain our intriguing result for cerebellar lobule V reserve which lost statistical significance in the final adjusted outcome model, particularly when contrasted with lobule VI.

Another interpretation could be that larger cerebellar brain volumes, both in the motor and in the cognitive domain, might increase the robustness of the cerebellum to atrophy and diaschisis to occur over time, particularly in more severely impaired patients with larger lesion loads.^54,55^ The extent of cerebellar diaschisis has been negatively correlated to improvement over time or functional gains under therapy, with positive and negative findings.^54,56,57^ The aspect of diaschisis and disconnection might also contribute to the explanation of why lobule V brain reserve lost statistically significance in the adjusted outcome models. This study has focussed on acute stroke patients with upper limb motor deficits, which might have biased the damage to the dentato-thalamo-cortical-tracts towards connections originating from lobule V and targeting hand representations of the primary motor cortex.^21,28,58^ Mechanistically, this might indicate that the contribution of cerebellar lobules to recovery processes, such as lobule V, might be limited by the extent to which their ascending fibre tracts are damaged by the stroke. Vice versa, one can also argue that innovative strategies by means of non-invasive cerebellar brain stimulation^59^ should target those cerebellar lobules of the motor and cognitive domain which show most preserved structural connectivity with the cortex. Importantly, longitudinal and high-resolution imaging would be needed to further investigate the influence of cerebellar volumes on courses of atrophy, characteristics of structural and functional cortico-cerebellar disconnections and clinical recovery from stroke over time.

There are several limitations to note. First, cerebellar volumes were considered as dichotomized binary variables indicating larger or smaller values than the median to increase the statistical power and to overcome the limitation of potential outliers and influential points. Together with LOOA and the sensitivity analysis, this approach guaranteed a high robustness of the findings despite the relatively small sample size. The arbitrary allocation of the individual patients to both groups might influence comparable analyses in independent samples. Second, statistical results were not corrected for multiple comparisons considering the exploratory nature of the study. Hence, the specificity is reduced, and further analyses on independent samples are required to further verify or falsify our results. Third, the present cohort consisted of patients with severe initial deficits. To what extent our findings will hold true for other cohorts, such as patients with moderate deficits remains to be determined. Fourth, detailed information about the type and intensity of neurorehabilitation between T_1_ and T_2_ was not available. These data could potentially influence the functional outcome after stroke. Future work is needed to address more specific questions regarding neurorehabilitation, e.g. whether cerebellar anatomy might impact the extent of treatment gains under therapy. Finally, the analyses were focused on cerebellar anatomy. Combined analyses of cerebellar anatomy together with other aspects of structural brain reserve, e.g. microstructure of various cortico-cortical or cortico-fugal motor pathways,^7^ whole-brain brain network characteristics^60,61^ including an assessment of the relationship between structural cerebellar reserve and functional cortico-cerebellar connectivity remain interesting topics for future studies on larger sample sizes.

## Abbreviations

BI =: Barthel index
ICV =: intracranial volume
LOOA =: leave-one-out model analysis
NIHSS =: National Institutes of Health Stroke Scale
MNI =: Montreal Institute of Neurology
MRS =: modified Rankin Scale
